# Real‐world outcomes of chemoradiotherapy for unresectable Stage III non‐small cell lung cancer: The SOLUTION study

**DOI:** 10.1002/cam4.3306

**Published:** 2020-07-30

**Authors:** Hidehito Horinouchi, Shinji Atagi, Satoshi Oizumi, Kadoaki Ohashi, Tomohiro Kato, Toshiyuki Kozuki, Masahiro Seike, Takashi Sone, Tomotaka Sobue, Takaaki Tokito, Hideyuki Harada, Tadashi Maeda, Tadashi Mio, Ikue Shirosaka, Kana Hattori, Eisei Shin, Haruyasu Murakami

**Affiliations:** ^1^ National Cancer Center Hospital Tokyo Japan; ^2^ National Hospital Organization Kinki‐Chuo Chest Medical Center Osaka Japan; ^3^ National Hospital Organization Hokkaido Cancer Center Hokkaido Japan; ^4^ Okayama University Hospital Okayama Japan; ^5^ National Hospital Organization Himeji Medical Center Hyogo Japan; ^6^ National Hospital Organization Shikoku Cancer Center Ehime Japan; ^7^ Nippon Medical School Hospital Tokyo Japan; ^8^ Kanazawa University Hospital Ishikawa Japan; ^9^ Graduate School of Medicine Osaka University Osaka Japan; ^10^ Kurume University Hospital Fukuoka Japan; ^11^ Shizuoka Cancer Center Shizuoka Japan; ^12^ National Hospital Organization Yamaguchi‐Ube Medical Center Yamaguchi Japan; ^13^ National Hospital Organization Kyoto Medical Center Kyoto Japan; ^14^ AstraZeneca K.K. Osaka Japan

**Keywords:** chemotherapy, clinical observations, lung cancer, radiation therapy

## Abstract

There are limited real‐world data on the treatment practices, outcomes, and safety of chemoradiotherapy (CRT) alone in potential candidates for immune checkpoint inhibitors (ICI) for unresectable non‐small cell lung cancer (NSCLC). In this study, we analyzed the safety and efficacy of CRT in patients who underwent CRT and would satisfy the key eligibility criteria for maintenance therapy with durvalumab (eg, no progression after CRT) in real‐world settings (m‐sub) for unresectable Stage III NSCLC between 1 January 2013 and 31 December 2015 at 12 sites in Japan. The m‐sub comprised 214 patients with a median follow‐up of 31.6 months (range 1.9‐65.8 months). Median overall survival (OS) and progression‐free survival (PFS) from completing CRT were 36.4 months (95% confidence interval [CI] 28.1 months to not reached) and 9.5 months (95% CI 7.7‐11.7 months), respectively. Consolidation chemotherapy did not influence OS or PFS. Median PFS was 16.9 vs 9.1 months in patients with vs without epidermal growth factor receptor (*EGFR*) mutations, with PFS rates of ~20% at 3‐4 years. Pneumonitis was the most common adverse event (according to MedDRA version 21.0J), and about half of events were grade 1. Pneumonitis mostly occurred 10‐24 weeks after starting CRT, peaking at 18‐20 weeks. Esophagitis and dermatitis generally occurred from 0 to 4 weeks, peaking at 2‐4 weeks after starting CRT. Pericarditis was rare and occurred sporadically. In conclusion, the results of the m‐sub provide real‐world insight into the outcomes of CRT, and will be useful for future evaluations of ICI maintenance therapy after CRT.

## Graphical abstract

We analyzed the efficacy and safety of chemoradiotherapy (CRT) for unresectable Stage III non‐small cell lung cancer in patients who would satisfy the key eligibility criteria for maintenance therapy with durvalumab (eg, no progression after CRT) in real‐world settings (m‐sub). Our results provide real‐world insight into the outcomes of CRT, and will be useful for future evaluations of immune checkpoint inhibitor (ICI) maintenance therapy after CRT.

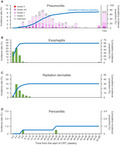

## 1 INTRODUCTION

Lung cancer is one of the most frequently diagnosed cancers with a high rate of cancer‐related deaths.^1^ About 30% of patients with non‐small cell lung cancer (NSCLC) present with Stage III disease, of which less than a third survive for longer than 5 years.^2^

Concurrent chemoradiotherapy (cCRT) with a curative intent is currently the standard treatment approach for unresectable Stage III NSCLC, and is recommended in the National Comprehensive Cancer Network (NCCN),^3^ European Society for Medical Oncology,^4^ a multidisciplinary consensus statement,^2^ and Japanese^5^ clinical guidelines. According to the Japanese guidelines,^5^ there is sufficient evidence from clinical trials to support the use of cisplatin + docetaxel and carboplatin + paclitaxel for the treatment of Japanese NSCLC patients. Other regimens, including cisplatin + vinorelbine and cisplatin + S‐1 (tegafur/gimeracil/oteracil) are also frequently used in the real world in Japan. However, there are currently limited data on how frequently these regimens are used and their outcomes in clinical practice.

Immune checkpoint inhibitors (ICIs) are a novel class of drugs for the treatment of NSCLC.^6^, ^7^, ^8^ ICIs activate the host's immune cells, especially T cells, to target specific tumor cells. ICIs include programmed cell death protein 1 (PD‐1), programmed death ligand 1 (PD‐L1), and cytotoxic T lymphocyte‐associated antigen 4 (CTLA‐4) inhibitors, and have revolutionized the NSCLC treatment landscape.^9^ Durvalumab, an IgG1κ monoclonal antibody that blocks the interaction between PD‐L1 with PD‐1 and CD80,^10^ was recently approved for maintenance therapy following radical CRT in patients with unresectable Stage III NSCLC in the absence of disease progression after CRT.^11^, ^12^

The approval of durvalumab for unresectable Stage III NSCLC was driven by the pivotal Phase III PACIFIC study, in which patients who met the study criteria after two or more cycles of platinum‐based CRT were randomized to either 10 mg/kg body weight durvalumab or placebo (2:1 ratio) every 2 weeks for up to 12 months as consolidation therapy.^13^ Durvalumab was associated with significantly longer progression‐free survival (PFS; 17.2 vs 5.6 months, *P* < .001).^14^ The benefit of durvalumab on overall survival (OS) relative to placebo persisted through to the most recent analysis at 36 months, with OS rates of 57.0% vs 43.5% (hazard ratio [HR] 0.69, 95% confidence interval [CI] 0.55‐0.86).^15^

In addition to evaluating the efficacy of ICIs, it is important to consider potential safety concerns. Pneumonitis was reported as a potential safety issue related to ICIs or the PD‐1/PD‐L1 axis (see eg^16^, ^17^, ^18^). Also, radiotherapy (RT) itself is acknowledged as a causative factor for pneumonitis (see eg^19^, ^20^, ^21^, ^22^, ^23^, ^24^). Accordingly, it is important to determine how frequently and when pneumonitis occurs during and after CRT for unresectable Stage III NSCLC.

In consideration of the efficacy and safety of durvalumab, the current NCCN guidelines recommend consolidation therapy with durvalumab following CRT for patients with unresectable Stage III NSCLC.^3^ Similarly, the updated Japanese guidelines also recommend consolidation therapy with durvalumab after cCRT.^25^

Currently, however, there are limited data on the outcomes and safety of CRT in real‐world settings. Real‐world data on existing therapies in patients who are likely to receive durvalumab or another ICI are needed in order to evaluate the contribution of ICIs to the outcomes and safety of CRT in real‐world settings. In addition, because pneumonitis is the most common adverse event (AE) associated with CRT itself, information on this AE is urgently needed, especially its timing of onset and severity.

We performed an observational, retrospective study (SOLUTION) of patients treated in the real world in Japan with the following objectives: (a) to collect data on the treatment patterns and outcomes of patients who underwent CRT before the approval of durvalumab or other ICIs; (b) to assess the outcomes of CRT in patients who would be eligible for durvalumab by applying the key eligibility criteria of the PACIFIC study^13^; and (c) to determine the safety profile of CRT, particularly pneumonitis and CRT‐induced interstitial lung disease.

## 2 MATERIALS AND METHODS

### 2.1 Ethics and consent

This study, SOLUTION, was performed in accordance with the principles of the Declaration of Helsinki and all applicable requirements in Japan, was approved by institutional review boards or ethics committees at participating sites, and registered on the University Hospital Medical Information Network database. Informed consent was obtained from surviving patients before data collection.

### 2.2 Study design, patients, and treatments

This observational, retrospective cohort study was performed at 12 sites distributed throughout Japan, and registered consecutive patients diagnosed with unresectable Stage III NSCLC between 1 January 2013 and 31 December 2015, who underwent platinum‐based CRT (Figure S1). Staging was based on the American Joint Committee on Cancer Staging Manual, version 7. All treatments, including consolidation chemotherapy, were at the clinician's discretion. Patients who had received any unapproved drugs as of 1 March 2018 (ie, before the approval of ICIs) or did not receive platinum‐based CRT were excluded. Patients who died or moved from the participating site were registered, providing this information was recorded and the patient or legal representative had an opportunity to opt out of the study. These patients were to be managed according to the instructions of the site's ethical review board. Data registration was performed between March 2018 and August 2018, and the database was locked in October 2018. It was planned to register ~300 patients, with a maximum of 30 patients per site to minimize bias. Consent was not obtained for nine patients. Data from registered patients were retrieved from the medical records.

Two analysis sets were defined: (a) the full analysis set (FAS) and (b) a subgroup of patients deemed eligible for maintenance therapy after CRT (m‐sub) (Figure S2). The FAS comprised all eligible patients. The m‐sub comprised patients who would now be deemed eligible for maintenance therapy after CRT in real‐world clinical practice by applying some of the key eligibility criteria from the PACIFIC study,^13^ namely: performance status of 0 or 1; completed ≥2 cycles of platinum‐based CRT with a therapeutic effect of complete response, partial response, or stable disease; and a radiation dose of 54‐66 Gy (Figure S1). Patients with any of the following were excluded from the m‐sub: carboplatin monotherapy, disease progression during primary treatment, grade ≥2 pneumonitis during primary treatment, administration of corticosteroids for pneumonitis of an unknown grade, or had not recovered from a grade ≥3 AE other than pneumonitis during primary treatment (Figure S1).

### 2.3 Endpoints

The primary efficacy endpoint was OS in the total population and was calculated from two timepoints: (a) the start of CRT and (b) from the completion of CRT. Secondary efficacy endpoints included OS and PFS in all patients and in subgroups of patients (FAS, m‐sub, CRT regimen, consolidation chemotherapy status, and epidermal growth factor receptor [*EGFR*] mutation status).

Safety was assessed in terms of treatment‐related AEs, with a special focus on pneumonitis/radiation pneumonitis and other CRT‐related AEs (esophagitis, radiation dermatitis, and pericarditis). Patients with these CRT‐related AEs were followed up for 2 years after the completion of primary treatment, including consolidation chemotherapy if performed. All AEs were recorded from the start of CRT, and were coded in terms of system organ class and preferred term using MedDRA version 21.0J. In patients with multiple AEs of the same type, only the highest grade AE was counted.

### 2.4 Statistical analyses

Patient, treatment characteristics, and safety data were assessed descriptively as the number (percent) of patients or median (range), as appropriate. OS and PFS were assessed using the Kaplan‐Meier method and were calculated from (a) the start of CRT and (b) completion of CRT for all patients and in subgroups (FAS, m‐sub, CRT regimen, consolidation chemotherapy status, and *EGFR* mutation status). The timing of onset of pneumonitis, assessed as the incidence rate in each 2‐week period up to 52 weeks and beyond 52 weeks from the start of CRT, and the cumulative incidence proportion over the follow‐up period were calculated using the FAS and m‐sub. HRs and 95% CIs were calculated as appropriate.

## 3 RESULTS

### 3.1 Patients

Of 318 patients initially registered, 12 were excluded for reasons given in Figure S2. Therefore, 306 patients were included in the FAS. Of these, 214 satisfied the eligibility criteria for inclusion in the m‐sub.

### 3.2 Characteristics and first‐line treatments of patients in the m‐sub

Tables 1 and 2 show the general characteristics and first‐line treatments for 214 patients in the m‐sub. Overall, 78.0% of patients were male and 86.9% had a current or past smoking history (Table 1). Approximately three‐quarters of patients had comorbidities, which included interstitial lung disease in one patient. Four patients had evidence of autoimmune disease. The m‐sub was equally divided into patients with Stage IIIA and Stage IIIB NSCLC. The most common histological types were adenocarcinoma and squamous cell carcinoma. The primary lesion was located in the right upper lobe in nearly half of the patients. Lung function parameters at baseline are shown in Table 1 for patients with available data.

**TABLE 1:** Patient characteristics in the m‐sub (N = 214)

	Value
Age, years, median (range)	65.0 (37‐79)
Sex	
Male	167 (78.0%)
Female	47 (22.0%)
Smoking history	
Current	67 (31.3%)
Past	119 (55.6%)
Never	28 (13.1%)
ECOG PS	
0	140 (65.4%)
1	74 (34.6%)
2	0 (0%)
3/4	0 (0%)
Comorbidities	
Yes	150 (70.1%)
Type of comorbidity	
COPD	38 (17.8%)
Autoimmune disease	4 (1.9%)
ILD	1 (0.5%)
IPF	0 (0%)
Non‐IPF	1 (0.5%)
Other	136 (63.6%)
Stage	
IIIA	107 (50.0%)
IIIB	107 (50.0%)
Histological type	
Adenocarcinoma	107 (50.0%)
Squamous cell carcinoma	79 (36.9%)
Neuroendocrine tumor	8 (3.7%)
Other	20 (9.3%)
Primary lesion location	
Right upper lobe	94 (43.9%)
Right middle lobe	8 (3.7%)
Right lower lobe	30 (14.0%)
Left upper lobe	68 (31.8%)
Left lower lobe	18 (8.4%)
%VC	n = 192
Mean (SD)	99.1 (19.2)
<80	30 (14.0%)
≥80	162 (75.7%)
FEV_1_/FVC, %	n = 192
Mean (SD)	70.3 (10.5)
<70	90 (42.1%)
≥70	102 (47.7%)
%DLco	n = 59
Mean (SD)	87.8 (24.9)
<70	18 (8.4%)
≥70	41 (19.2%)
SpO_2_, %	n = 211
Mean (SD)	97.1 (1.4)
<90	0 (0%)
≥90	211 (98.6%)
Reason for terminating first‐line RT	
Completed as planned	212 (99.1%)
PD	0 (0%)
Toxicity	0 (0%)
Other	2 (0.9%)
Follow‐up period, days	
Mean (SD)	980.9 (547.0)
Median (range)	963.0 (57‐2002)

Note

Corresponding data in the full analysis set are presented in Table S1.

Values are number (%) of patients unless otherwise stated.

Abbreviations: %DLco, percent of diffusion capacity; %VC, percent of vital capacity; COPD, chronic obstructive pulmonary disease; ECOG, Eastern Cooperative Oncology Group; FEV_1_, forced expiratory volume in 1 second; FVC, forced vital capacity; ILD, interstitial lung disease; IPF, idiopathic pulmonary fibrosis; m‐sub, subgroup of patients deemed eligible for maintenance therapy after chemoradiotherapy; PD, progressive disease; PS, performance status; RT, radiotherapy; SD, standard deviation; SpO_2_, oxygen saturation.

**TABLE 2:** First‐line treatment regimens in the m‐sub (N = 214)

Chemotherapy	Value^a^	RT	Value
Cisplatin + vinorelbine	76 (35.5%)	Dose	n = 214
Cisplatin + docetaxel	50 (23.4%)	Median (range), Gy	60.0 (54.0‐66.0)
Carboplatin + paclitaxel	44 (20.6%)	<54 Gy	0 (0%)
Cisplatin + S‐1	32 (15.0%)	≥54 to ≤66 Gy	214 (100.0%)
Carboplatin	0 (0.0%)	>66 Gy	0 (0%)
Other	12 (5.6%)	V20	n = 210
Carboplatin + S‐1	6 (2.8%)	Median (range)	23.20% (1.6%‐41.6%)
Carboplatin + pemetrexed	2 (0.9%)	<25%	—
Cisplatin + pemetrexed	2 (0.9%)	≥25%	—
Cisplatin + etoposide	2 (0.9%)	<35%	202 (94.4%)
		≥35%	8 (3.7%)
		V5	n = 210
		Median (range)	36.45% (4.0%‐69.4%)
		<65%	209 (97.7%)
		≥65%	1 (0.5%)

Note

Corresponding data in the full analysis set are presented in Table S2.

Abbreviations: m‐sub, subgroup of patients deemed eligible for maintenance therapy after CRT; RT, radiotherapy; S‐1, tegafur/gimeracil/oteracil; V20, volume of lung that received a dose of ≥20 Gy; V5, volume of lung that received a dose of ≥5 Gy.

^a^ Values are number (%) of patients who received each chemotherapy regimen in the total population.

The most common first‐line chemotherapy regimens were cisplatin + vinorelbine in 76 (35.5%) patients and cisplatin + docetaxel in 50 (23.4%) patients (Table 2). All of the patients completed their first‐line chemotherapy regimen. The RT dose was ≥54 to <66 Gy in all of the patients (per eligibility criteria), with a V20 (volume of lung parenchyma that received ≥20 Gy^24^) of <35% in 94.4% of patients and V5 (volume of lung parenchyma that received ≥5 Gy^24^) of <65% in 97.7% (Table 2). RT was terminated in two patients (both at the patient's request) (Table 1).

The characteristics and first‐line treatments of patients included in the FAS are presented in Tables S1 and S2, respectively.

### 3.3 OS and PFS in the m‐sub and FAS

**FIGURE 1:**
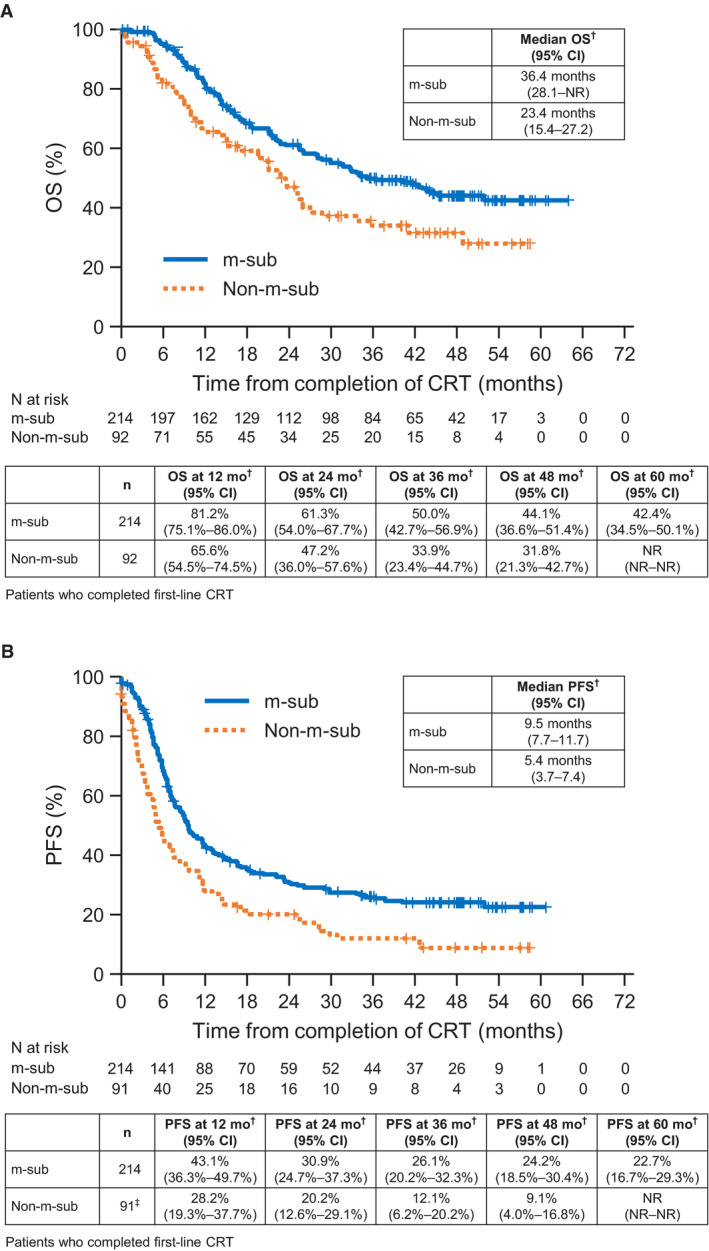
Kaplan‐Meier plots of overall survival (A) and progression‐free survival (B) from the completion of chemoradiotherapy in the m‐sub and non‐m‐sub for all patients who completed first‐line therapy. CI, confidence interval; CRT, chemoradiotherapy; mo, month; m‐sub, subgroup of patients deemed eligible for maintenance therapy after CRT; non‐m‐sub, patients from the full analysis set excluded from the m‐sub; NR, not reached; OS, overall survival; PFS, progression‐free survival. ^†^Kaplan‐Meier estimated values; ^‡^n = 91 because PFS could not be determined in one patient in the non‐m‐sub

The median follow‐up of the m‐sub was 31.6 months (range 1.9‐65.8 months). Figure 1 shows the OS and PFS calculated from the completion of CRT for the m‐sub and the non‐m‐sub (92 patients from the FAS excluded from the m‐sub). The estimated median OS and PFS in the m‐sub were 36.4 months (95% CI 28.1 months to not reached) and 9.5 months (95% CI 7.7‐11.7 months), respectively. The OS and PFS rates at 36 months were 50.0% and 26.1%, respectively. These values are similar to those in the FAS (N = 306; Figure S3), for which the estimated median OS and PFS from the start of CRT were 33.1 months (95% CI 27.0‐42.5 months) and 10.3 months (95% CI 9.1‐11.9 months), respectively.

### 3.4 OS and PFS in subgroups of patients

#### 3.4.1 Outcomes according to the chemotherapy regimen

OS and PFS measured from the start of CRT were also compared between subgroups of patients according to the chemotherapy regimen (Figure S4). Median OS from the start of CRT was longest with cisplatin + vinorelbine and carboplatin + paclitaxel, and OS was also good with cisplatin + docetaxel and cisplatin + vinorelbine after more than 3 years (Figure S4A). PFS tended to be longer in patients who received cisplatin with either docetaxel or vinorelbine (Figure S4B). OS and PFS were shortest in patients who received carboplatin alone, with a survival rate of 25.4% at 36 months and median PFS of 6.8 months.

#### 3.4.2 Outcomes according to *EGFR* mutation status

**FIGURE 2:**
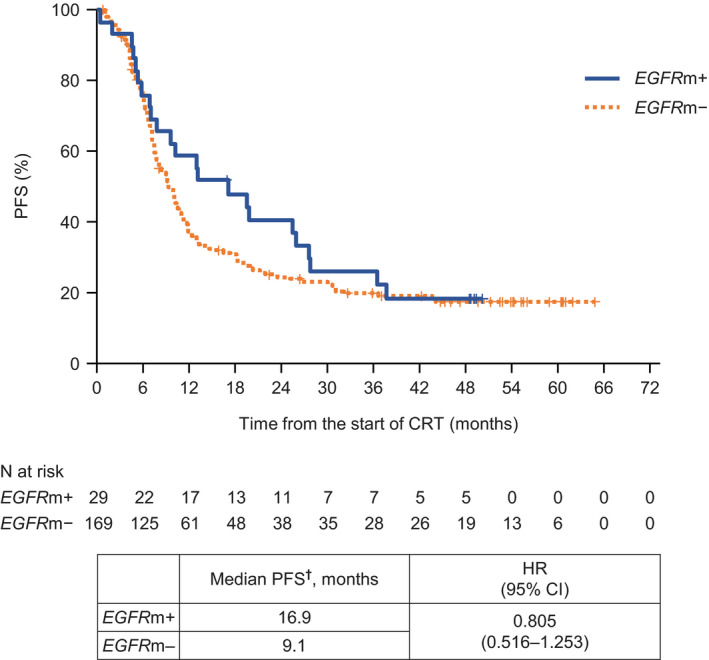
Kaplan‐Meier plot of progression‐free survival according to *EGFR* mutation status. CI, confidence interval; CRT, chemoradiotherapy; *EGFR*m, epidermal growth factor receptor mutation; HR, hazard ratio; PFS, progression‐free survival. ^†^Actual values

**FIGURE 3:**
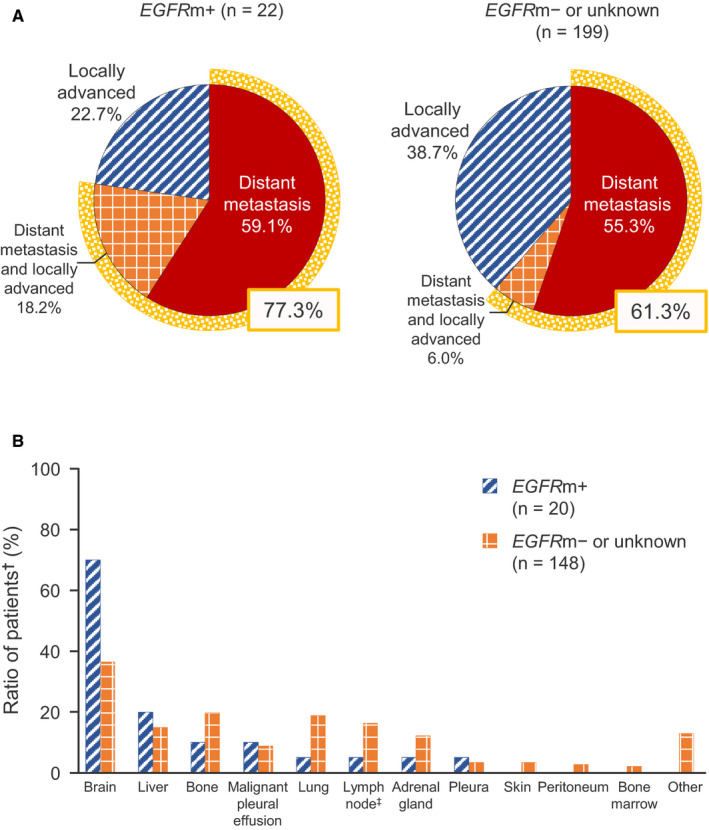
Pattern of disease progression (A) and sites of distant metastasis (B) according to *EGFR* mutation status. *EGFR*m, epidermal growth factor receptor mutation. ^†^The denominator is the number of patients with distant metastasis. ^‡^Distant lymph nodes (not regional lymph nodes)

The *EGFR* mutation status was assessed in 199 patients, of which 29 had *EGFR* mutations (*EGFR*m+). Median PFS was 16.9 and 9.1 months in the *EGFR*m+ and *EGFR*m− groups of patients, respectively (HR 0.805, 95% CI 0.516‐1.253; Figure 2). In both groups, the PFS rates were about 20% at 3‐4 years, with no additional cases of disease progression during this period of time. Distant metastasis, with or without local progression, was the main cause of disease progression, occurring in 77.3% of *EGFR*m+ patients and 61.3% of *EGFR*m− patients (Figure 3A), while locally advanced disease without distant metastasis was found in 22.7% and 38.7%, respectively. Among 20 *EGFR*m+ patients with distant metastases, the brain was the main site, accounting for 70.0% of cases with distant metastasis, followed by the liver in 20.0%. Among 148 *EGFR*m−/unknown patients with distant metastases, brain metastases were found in 36.5%, with bone, lung, and lymph node metastases in 19.6%, 18.9%, and 16.2% of patients, respectively (Figure 3B). Of 22 *EGFR*m+ patients with confirmed progression, 15 (68.2%) were subsequently treated with an EGFR tyrosine kinase inhibitor.

#### 3.4.3 Effects of consolidation chemotherapy on OS and PFS

We next compared the outcomes between patients who did or did not receive consolidation chemotherapy. The general characteristics were broadly comparable, except for a longer median follow‐up (970.5 vs 749.0 days) in patients who received consolidation chemotherapy than in patients who did not. As shown in Figure S5, consolidation chemotherapy did not significantly contribute to OS or PFS in the FAS.

### 3.5 Safety

#### 3.5.1 Overall safety (FAS)

Safety was assessed in terms of the frequency and grade of AEs that occurred any time from the start of CRT. AEs occurred in 99.0% of patients in the FAS, with grade 1, 2, 3, 4, and 5 AEs in 10.8%, 31.0%, 38.2%, 15.7%, and 0.7% of patients, respectively. Table 3 lists the AEs by grade that occurred in ≥5% of patients. Pneumonitis (72.9%) and esophagitis (59.5%) were the two most common types of AEs.

**TABLE 3:** Adverse events by type and grade in ≥5% of patients in the full analysis set (N = 306)

AE (MedDRA 21.0J)	Any grade	Grade 1	Grade 2	Grade 3	Grade 4	Grade 5	Unknown grade
Any AE	303 (99.0%)	33 (10.8%)	95 (31.0%)	117 (38.2%)	48 (15.7%)	2 (0.7%)	8 (2.6%)
Pneumonitis	223 (72.9%)	123 (40.2%)	52 (17.0%)	20 (6.5%)	0 (0%)	1 (0.3%)	27 (8.8%)
AEs other than pneumonitis	293 (95.8%)	26 (8.5%)	96 (31.4%)	110 (35.9%)	48 (15.7%)	2 (0.7%)	11 (3.6%)
Esophagitis	182 (59.5%)	72 (23.5%)	79 (25.8%)	15 (4.9%)	1 (0.3%)	0 (0%)	15 (4.9%)
WBC count decreased	146 (47.7%)	8 (2.6%)	44 (14.4%)	63 (20.6%)	31 (10.1%)	0 (0%)	0 (0%)
Decreased appetite	107 (35.0%)	60 (19.6%)	27 (8.8%)	16 (5.2%)	0 (0%)	0 (0%)	4 (1.3%)
Radiation dermatitis	100 (32.7%)	67 (21.9%)	27 (8.8%)	3 (1.0%)	0 (0%)	0 (0%)	3 (1.0%)
Nausea	81 (26.5%)	43 (14.1%)	28 (9.2%)	9 (2.9%)	0 (0%)	0 (0%)	1 (0.3%)
Constipation	72 (23.5%)	46 (15.0%)	24 (7.8%)	1 (0.3%)	0 (0%)	0 (0%)	1 (0.3%)
Malaise	71 (23.2%)	53 (17.3%)	18 (5.9%)	0 (0%)	0 (0%)	0 (0%)	0 (0%)
Neutrophil count decreased	68 (22.2%)	4 (1.3%)	14 (4.6%)	32 (10.5%)	17 (5.6%)	0 (0%)	1 (0.3%)
Platelet count decreased	52 (17.0%)	26 (8.5%)	22 (7.2%)	3 (1.0%)	1 (0.3%)	0 (0%)	0 (0%)
Anemia	50 (16.3%)	16 (5.2%)	20 (6.5%)	14 (4.6%)	0 (0%)	0 (0%)	0 (0%)
Pyrexia	45 (14.7%)	37 (12.1%)	4 (1.3%)	2 (0.7%)	1 (0.3%)	0 (0%)	1 (0.3%)
Diarrhea	40 (13.1%)	21 (6.9%)	14 (4.6%)	5 (1.6%)	0 (0%)	0 (0%)	0 (0%)
Hiccup	39 (12.7%)	20 (6.5%)	13 (4.2%)	2 (0.7%)	0 (0%)	0 (0%)	4 (1.3%)
Vomiting	23 (7.5%)	15 (4.9%)	5 (1.6%)	1 (0.3%)	0 (0%)	0 (0%)	2 (0.7%)
Stomatitis	16 (5.2%)	10 (3.3%)	6 (2.0%)	0 (0%)	0 (0%)	0 (0%)	0 (0%)
Alopecia	16 (5.2%)	14 (4.6%)	2 (0.7%)	0 (0%)	0 (0%)	0 (0%)	0 (0%)

Note

Values are number (%) of patients.

Abbreviations: AE, adverse event; WBC, white blood cell.

#### 3.5.2 CRT‐related adverse events in the m‐sub and FAS

**FIGURE 4:**
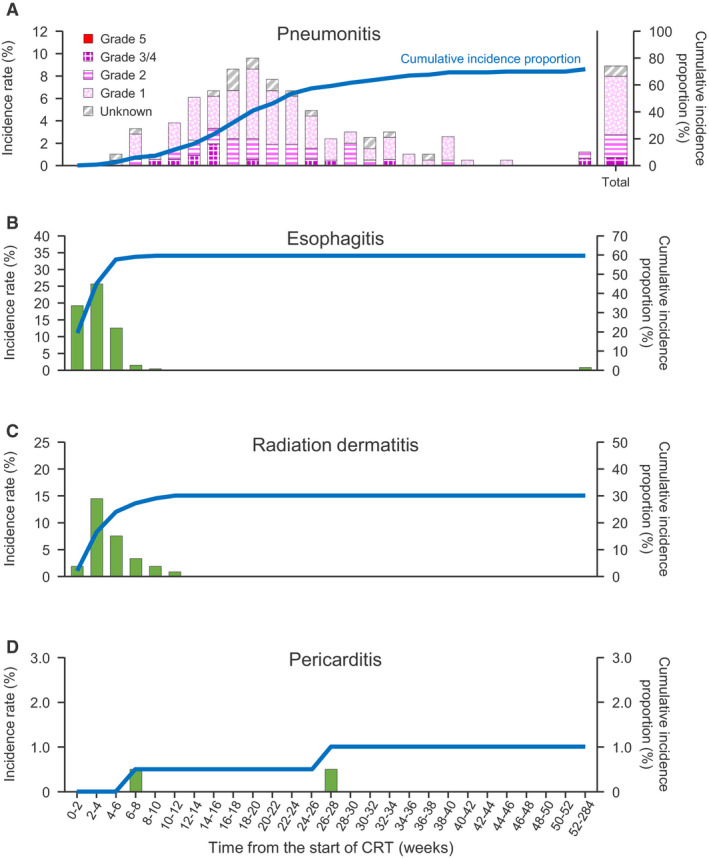
Timing of onset and cumulative incidence rate of pneumonitis (A), esophagitis (B), radiation dermatitis (C), and pericarditis (D) in the m‐sub. The grade of pneumonitis is also indicated. Timing of onset was measured from the start of CRT. CRT, chemoradiotherapy; m‐sub, subgroup of patients deemed eligible for maintenance therapy after CRT

Patients with pneumonitis, esophagitis, radiation dermatitis, and pericarditis were followed up for 2 years. The timing of onset and incidence from the start of CRT in the m‐sub is shown in Figure 4. Pneumonitis was the most common AE in the m‐sub, and about half of all episodes were classified as grade 1 (Figure 4A). There were no episodes of grade 5 pneumonitis in the m‐sub; grade 5 pneumonitis occurred in one patient in the non‐m‐sub. Most episodes of pneumonitis occurred about 10‐24 weeks after the start of CRT, with a peak onset at around 18‐20 weeks (Figure 4A), which is several weeks after the completion of CRT and would coincide with the start of durvalumab following first‐line therapy. Esophagitis and radiation dermatitis mostly occurred within 0‐4 weeks of starting CRT, with a peak at 2‐4 weeks. Cases of pericarditis were sporadic (Figure 4B‐D). The timing of onset and cumulative incidence rates of pneumonitis, esophagitis, radiation dermatitis, and pericarditis in the FAS are shown in Figure S6, and are essentially similar to those observed in the m‐sub. We also assessed possible risk factors for pneumonitis in the FAS, and found possible associations with RT V20, V5, and primary tumor site (Table S3).

#### 3.5.3 Safety according to the chemotherapy regimen and consolidation chemotherapy (FAS)

Pneumonitis was the most frequent AE regardless of the chemotherapy regimen used (Table S4). The grade of pneumonitis according to the chemotherapy regimen used is indicated in Figure S7A. These data suggest that pneumonitis of grade ≥2 was more frequent in patients administered carboplatin + paclitaxel or other chemotherapy regimens than in patients administered cisplatin + vinorelbine or cisplatin + docetaxel. Other common AEs included esophagitis, white blood cell count decreased, decreased appetite, and radiation dermatitis. A similar pattern was observed in the m‐sub (Figure S7B).

We also analyzed the AEs according to whether or not consolidation chemotherapy was performed (Table S5). The rates of individual AEs were generally similar between patients who did or did not receive consolidation chemotherapy. However, white blood cell count decreased (54.1% vs 42.4%), decreased appetite (41.2% vs 29.7%), nausea (35.8% vs 21.5%), and constipation (32.4% vs 15.2%) were more frequent among patients who received consolidation chemotherapy. There were some differences in the types of AEs among the first‐line chemotherapy regimens and between patients who did and did not receive consolidation chemotherapy (data not shown).

## 4 DISCUSSION

This study was performed to collate information on the treatment practices, outcomes of each treatment pattern, and safety of radical CRT in patients with unresectable Stage III NSCLC in a real‐world setting. In this study, we focused on a cohort of 214 patients who would be deemed potential candidates for maintenance therapy (m‐sub) based on the key eligibility criteria of the PACIFIC study, namely, no disease progression after CRT, performance status of 0‐1, a total radiation dose of 54‐66 Gy, and no grade ≥2 pneumonitis. The median OS and PFS calculated from the completion of CRT were 36.4 months (95% CI 28.1 months to not reached) and 9.5 months (95% CI 7.7‐11.7 months), respectively. In addition, we found that consolidation chemotherapy did not have a significant contribution to OS; *EGFR* mutation status did not have a significant impact on PFS in patients who underwent CRT; the 3‐year OS rate was better with cisplatin‐based regimens; and the incidence of pneumonitis was lowest with cisplatin + vinorelbine. Regarding safety, pneumonitis was the most frequent AE during CRT, with a peak incidence at 10‐24 weeks after the start of CRT.

The median PFS and OS in the m‐sub can be discussed in the context of results from the Japanese subset of patients in the placebo group of the PACIFIC study. In those patients, the median PFS (from randomization) was 7.2 months and median OS was not reached (unpublished data); the corresponding values in the m‐sub in our study were 9.5 and 36.4 months. If we take into account the differences in the starting point used to calculate PFS and OS, the outcomes in the m‐sub and the Japanese subset in PACIFIC appear to be comparable. Although the present results were based on data from 12 selected sites, these findings also suggest that the results from the placebo group in PACIFIC reflect real‐world observations in Japan.

We also investigated the types of first‐line chemotherapies. The NCCN guidelines for NSCLC currently recommend several regimens for cCRT, including cisplatin + etoposide, cisplatin + vinblastine, carboplatin + pemetrexed, or carboplatin + paclitaxel.^3^ By comparison, the Japanese clinical guidelines recommend the use of carboplatin + paclitaxel, cisplatin + docetaxel, or carboplatin alone, in combination with RT.^25^ Consistent with the Japanese guidelines, these regimens were frequently used in our cohort, although additional regimens, such as cisplatin + vinorelbine or S‐1, were also used. This analysis revealed some differences in the prognosis of patients depending on the chemotherapy regimen used, as OS and PFS tended to be better in patients who received cisplatin + vinorelbine. Further studies are needed to understand whether these differences are related to the type of chemotherapy used or other factors that may influence the selection of chemotherapy.

Assessment of the outcomes of first‐line CRT in patients with and without *EGFR* mutations revealed that, although the median PFS was longer in the *EGFR*m+ group, the HR was 0.805 and the 3/4‐year PFS rate was ~20% regardless of *EGFR* mutation status. Other studies have also assessed the impact of *EGFR* mutations on treatment outcomes.^26^, ^27^ Ochiai et al^26^ found no differences in the objective response rate (odds ratio 1.46, 95% CI 0.79‐2.70) or the disease recurrence rate (odds ratio 1.37, 95% CI 0.68‐2.75) after CRT between the *EGFR*m+ and *EGFR* wild‐type groups. On the other hand, Tanaka et al^27^ reported that PFS was significantly shorter in the *EGFR*m+ group and that the ratio of distant metastasis in patients with disease recurrence was relatively higher in this group than the *EGFR* wild‐type group. We also observed a higher ratio of distant metastasis in the *EGFR*m+ group than the *EGFR*m−/unknown group. Based on these findings, a treatment strategy for controlling distant metastasis, especially brain metastasis, may need to be considered.

We also evaluated the impact of consolidation chemotherapy on OS and PFS because there is little real‐world information in this setting. In our study, the median OS and PFS were similar between patients who did and did not receive consolidation chemotherapy. These findings essentially mimic those of a pooled analysis of 41 Phase II/III studies involving patients with locally advanced NSCLC, published before 31 December 2011.^28^ In that analysis, the pooled median OS was 19.0 months (95% CI 17.3‐21.0 months) in patients who received consolidation therapy vs 17.9 months (95% CI 16.1‐19.9 months) in patients who did not, with a predicted HR of 0.94 (95% CI 0.81‐1.09, *P* = .40). Our results also suggest that consolidation chemotherapy, even with third‐generation anticancer drugs, following CRT does not contribute to the improvement of OS in patients with unresectable Stage III NSCLC.

Another objective of this study was to elucidate the safety of CRT in real‐world settings. As anticipated, the safety profile was dominated by CRT‐related AEs, especially pneumonitis. Interestingly, the incidence of pneumonitis peaked at about 18‐20 weeks after the start of first‐line therapy or about 10‐12 weeks after completing CRT. This indicates that pneumonitis has a delayed onset and may be infrequent during CRT. Instead, pneumonitis may be more likely to occur coincidentally at the initiation of consolidation chemotherapy. This may raise the question of whether the clinical reports on pneumonitis during durvalumab therapy are due to the drug itself or represent a delayed effect of CRT. In the PACIFIC study, pneumonitis/radiation pneumonitis occurred in about one‐quarter of patients in the placebo group (24.8%). Although the frequency of pneumonitis/radiation pneumonitis of any grade was higher in the durvalumab group (33.9%), the frequency of grade 3/4 events was comparable between the durvalumab and placebo groups (3.4% vs 2.6%). The incidence of pneumonitis was 73.6% in Japanese patients enrolled in the PACIFIC study (unpublished data), similar to that in the present study (74.3% in the m‐sub). To our knowledge, our study was the first to investigate the frequency and the onset timing of pneumonitis during/after CRT in real‐world settings.

Other CRT‐related AEs included esophagitis and radiation dermatitis, which mostly occurred within 8‐10 weeks of starting CRT, with few additional events thereafter. Other studies have also reported the types of AEs occurring after RT for NSCLC.^29^, ^30^ In elderly patients, lung‐related (6.5%) and esophagus‐related (1.1%) AEs were among the most frequent grade 3/4 AEs occurring ≥90 days after CRT, while lung‐related (5.3%) and heart‐related (2.1%) grade 3/4 AEs were more frequent after RT.^29^ In another study of 125 patients with Stage II‐III NSCLC, of which 27% had preexisting cardiac disease and 84% underwent CRT, 19 patients experienced a grade ≥3 cardiac event at a median of 11 months (range 0.4‐63 months) after treatment.^30^ In the present study, there were few cardiac events, and pericarditis occurred sporadically in two (0.7%) patients.

Possible limitations of this study include its retrospective design, which may have distorted the results because of unmeasured confounders, but attempts were made to minimize bias, including the registration of consecutive patients. In addition, our study involved only 12 Japanese sites, and the sample size was limited. Further investigations with a greater sample size are needed to confirm our results.

In conclusion, this study provided valuable, clinically relevant data regarding the outcomes and safety of CRT in real‐world settings. In particular, we found that OS and PFS differed slightly among first‐line chemotherapy regimens; consolidation therapy did not have a significant contribution to OS or PFS; and that the median PFS was 16.9 vs 9.1 months in *EGFR*m+ patients vs *EGFR*m–/unknown patients, with a 3/4‐year PFS rate of ~20% regardless of *EGFR* mutation status. In terms of safety, we found that pneumonitis was the most frequent AE and the peak incidence of pneumonitis coincided with the period of durvalumab maintenance therapy, suggesting that pneumonitis and other AEs may be due to the effects of prior CRT rather than maintenance therapy with durvalumab. The results using the m‐sub group provide valuable real‐world evidence that can be referenced in future evaluations of ICI therapy after CRT in real‐world clinical practice.

## CONFLICT OF INTEREST

Hidehito Horinouchi reports lecture fees, honoraria, or other fees from Eli Lilly, AstraZeneca, MSD, Ono, and Bristol‐Myers Squibb, and research grants from MSD, Chugai, Ono, Bristol‐Myers Squibb, AstraZeneca, Daiichi‐Sankyo, Novartis, and Genomic Health. Shinji Atagi reports research grants from AstraZeneca, MSD, Eli Lilly, Chugai, Ono, Taiho, Boehringer Ingelheim, Pfizer, and Bristol‐Myers Squibb. Satoshi Oizumi reports lecture fees, honoraria, or other fees from AstraZeneca and Eli Lilly, and research grants from AstraZeneca, Bristol‐Myers Squibb, Chugai, Kyowa Kirin, Ono, Merck Biopharma, Taiho, and Pfizer. Kadoaki Ohashi reports research grants from Boehringer Ingelheim, AstraZeneca, Eli Lilly, and Novartis. Toshiyuki Kozuki reports lecture fees, honoraria, or other fees from Chugai, AstraZeneca, and Eli Lilly. Hideyuki Harada reports lecture fees, honoraria, or other fees from AstraZeneca. Ikue Shirosaka, Kana Hattori, and Eisei Shin are employees of AstraZeneca. Haruyasu Murakami reports lecture fees, honoraria, or other fees from AstraZeneca and Chugai. Tomohiro Kato, Masahiro Seike, Takashi Sone, Tomotaka Sobue, Takaaki Tokito, Tadashi Maeda, and Tadashi Mio declare no conflict of interest. AstraZeneca was involved in the design and conduct of the study, statistical analyses, and in writing of the report. The authors had full access to the data.

## AUTHOR CONTRIBUTIONS

Hideyuki Harada, Ikue Shirosaka, Eisei Shin, and Haruyasu Murakami conceived the study. Hidehito Horinouchi, Tomotaka Sobue, Hideyuki Harada, Ikue Shirosaka, Eisei Shin, and Haruyasu Murakami designed the study. Hidehito Horinouchi, Shinji Atagi, Satoshi Oizumi, Kadoaki Ohashi, Tomohiro Kato, Toshiyuki Kozuki, Masahiro Seike, Takashi Sone, Takaaki Tokito, Hideyuki Harada, Tadashi Maeda, Tadashi Mio, and Haruyasu Murakami contributed to data collection. Ikue Shirosaka and Kana Hattori analyzed the data. Hidehito Horinouchi, Tomotaka Sobue, Hideyuki Harada, Ikue Shirosaka, Kana Hattori, Eisei Shin, and Haruyasu Murakami wrote the first draft. All authors contributed to data interpretation, revised the manuscript, approved the final draft, and take accountability for the accuracy and integrity of the work.

## Supporting information

Fig S1‐S7

Table S1‐S5

## Data Availability

Data underlying the findings described in this study may be obtained in accordance with AstraZeneca's data sharing policy described at https://astrazenecagrouptrials.pharmacm.com/ST/Submission/Disclosure.
