# Supplementary material for: Real‐world outcomes of chemoradiotherapy for unresectable Stage III non‐small cell lung cancer: The SOLUTION study
Source: Cancer Med. 2020 Jul 30;9(18):6597–608. doi: 10.1002/cam4.3306 (PMC7520333; doi:10.1002/cam4.3306)
Supplement: Supplementary file 1 — Fig S1‐S7 [file CAM4-9-6597-s001.pdf]

FIGURE S1 Patient eligibility and study design

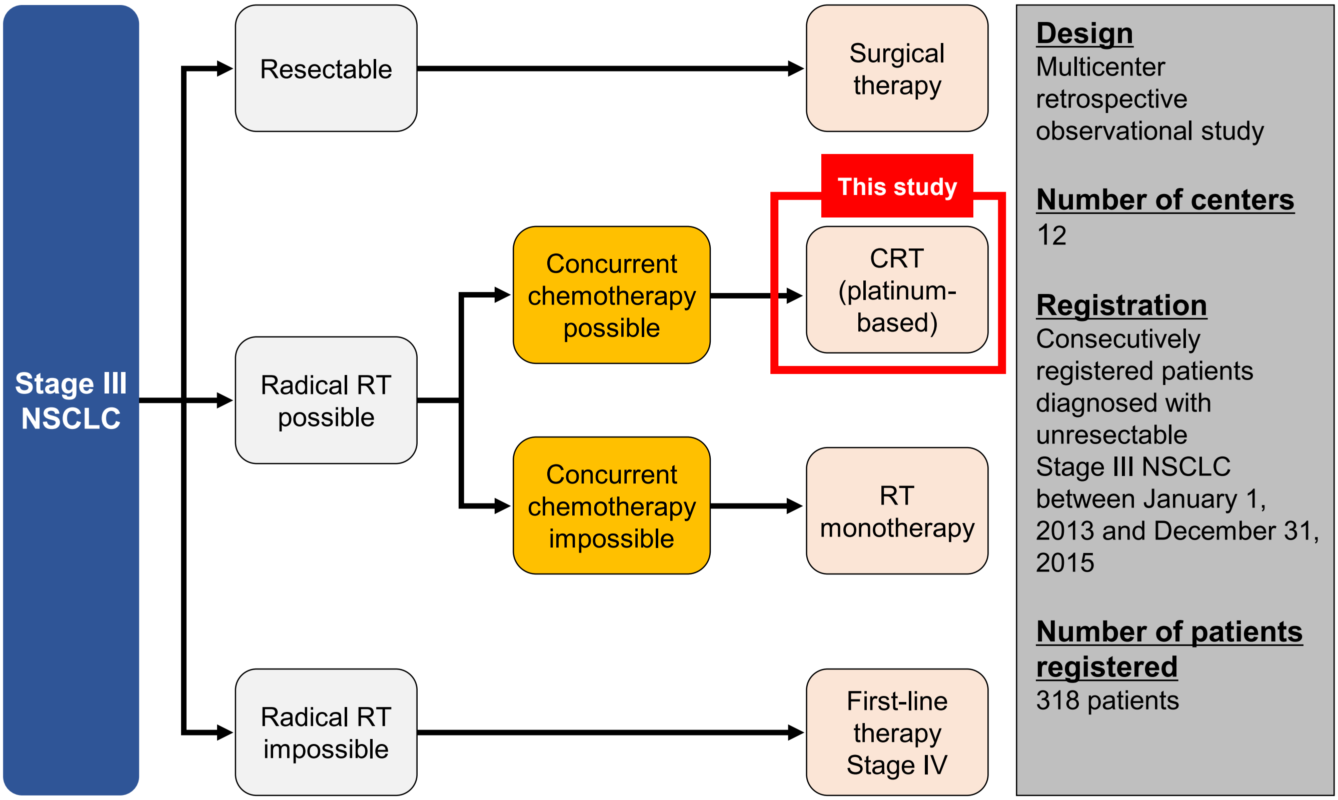

Eligibility criteria for the m-sub population†

- At least two cycles of platinum-based chemotherapy concurrent with definitive radiotherapy
- Performance status 0 or 1
- Complete response, partial response, or stable disease on first-line CRT and no progression at completion of CRT
- Total radiation dose of 54–66 Gy

Exclusion criteria for the m-sub population†

- Carboplatin monotherapy
- Grade ≥3 treatment-related adverse event (other than pneumonitis) during first-line therapy, unless the patient had recovered prior to enrolment
- Grade ≥2 pneumonitis during the first-line therapy or pneumonitis of unknown grade treated with a steroid

Abbreviations: NSCLC, non-small cell lung cancer; RT, radiotherapy; CRT, chemoradiotherapy; m-sub, subgroup of patients deemed eligible for maintenance therapy after CRT.

†Based on some of the main eligibility criteria from the PACIFIC study [Antonia SJ, Villegas A, Daniel D, et al. *N Engl J Med*. 2017;377:1919-1929].

FIGURE S2 Patient flow chart

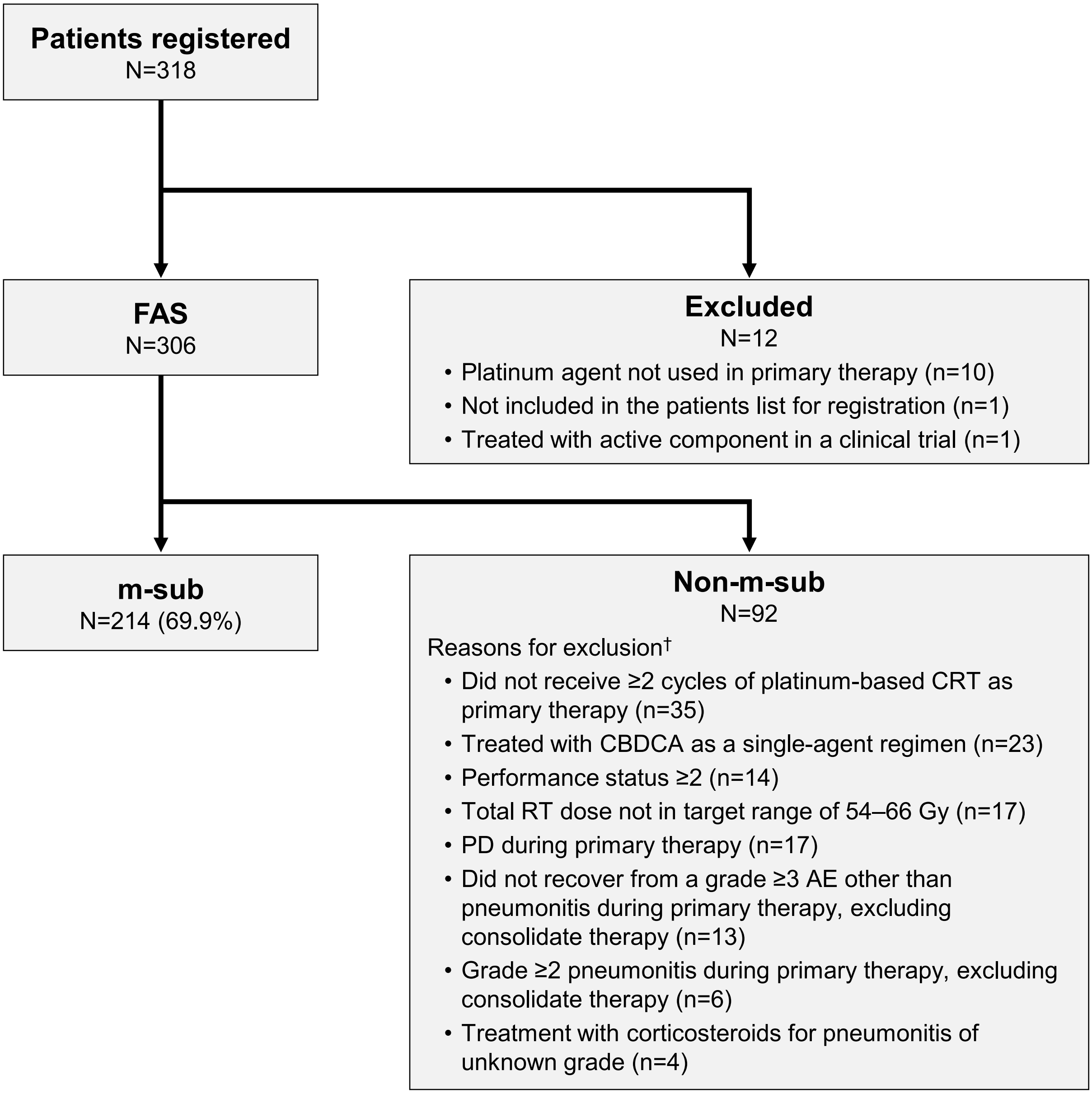

Abbreviations: FAS, full analysis set; m-sub, subgroup of patients deemed eligible for maintenance therapy after CRT; non-m-sub, patients from the FAS excluded from the m-sub; CRT, chemoradiotherapy; CBDCA, carboplatin; RT, radiotherapy; PD, progressive disease; AE, adverse event.

†Multiple reasons may apply.

FIGURE S3 Kaplan–Meier plots of overall survival (A) and progression-free survival (B) from the start of chemoradiotherapy in the full analysis set

A

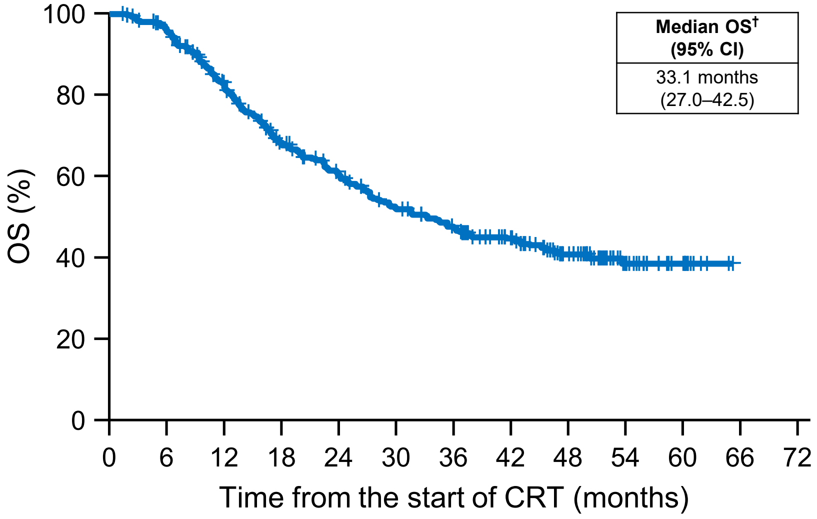

N at risk	306	285	236	184	156	130	113	89	60	28	13	0	0

n	OS at 12 mo† (95% CI)	OS at 24 mo† (95% CI)	OS at 36 mo† (95% CI)	OS at 48 mo† (95% CI)	OS at 60 mo† (95% CI)
306	82.9% (78.1%–86.8%)	60.6% (54.6%–66.1%)	47.4% (41.3%–53.3%)	40.4% (34.2%–46.5%)	38.3% (31.8%–44.8%)

B

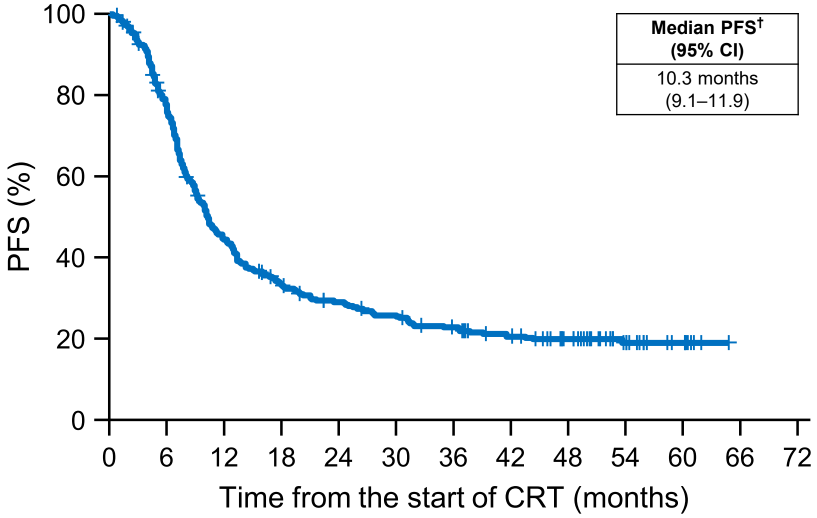

N at risk	305	229	130	96	79	69	58	48	35	18	8	0	0

n	PFS at 12 mo† (95% CI)	PFS at 24 mo† (95% CI)	PFS at 36 mo† (95% CI)	PFS at 48 mo† (95% CI)	PFS at 60 mo† (95% CI)
306	44.4% (38.7%–50.0%)	29.1% (24.0%–34.4%)	22.7% (18.1%–27.8%)	19.8% (15.3%–24.7%)	18.8% (14.2%–23.9%)

Abbreviations: OS, overall survival; CRT, chemoradiotherapy; mo, month; CI, confidence interval; PFS, progression-free survival

†Kaplan-Meier estimated values.

FIGURE S4 Kaplan–Meier plots of overall survival (A) and progression-free survival (B) from the start of chemoradiotherapy according to the chemotherapy regimen in the full analysis set

A

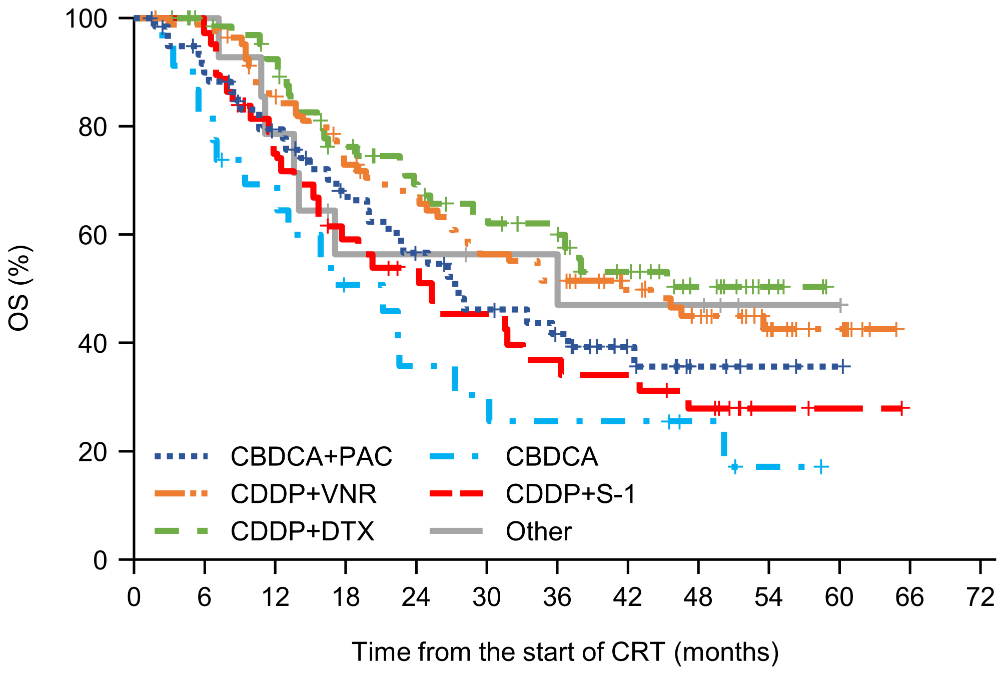

N at risk													
CBDCA+PAC	62	53	44	35	28	22	18	11	4	2	1	0	0
CDDP+VNR	92	90	76	63	56	47	43	34	26	16	9	0	0
CDDP+DTX	71	66	59	46	39	33	28	22	13	5	0	0	0
CBDCA	23	19	15	10	7	6	5	5	3	1	0	0	0
CDDP+S-1	44	43	31	23	19	16	13	12	9	2	1	0	0
Other	14	14	11	7	7	6	6	5	5	2	2	0	0

	**CBDCA+PAC** n=62	**CDDP+VNR** n=92	**CDDP+DTX** n=71	**CBDCA** n=23	**CDDP+S-1** n=44	**Other** n=14
**Median OS**† **(95% CI)**	27.6 months (19.9–42.5)	41.5 months (27.0–NR)	NR (30.0–NR)	21.2 months (9.4–30.1)	25.2 months (15.6–36.3)	36.0 months (11.1–NR)
OS at 12 months† (95% CI)	79.5% (66.7%–87.8%)	85.5% (76.4%–91.3%)	92.3% (82.4%–96.7%)	69.3% (46.1%–84.0%)	76.7% (60.9%–86.7%)	78.6% (47.2%–92.5%)
OS at 24 months† (95% CI)	56.6% (42.4%–68.6%)	66.9% (56.0%–75.7%)	69.3% (56.1%–79.2%)	35.6% (16.4%–55.3%)	54.1% (37.8%–67.9%)	56.3% (27.2%–77.6%)
OS at 36 months† (95% CI)	41.8% (28.2%–54.8%)	51.4% (40.4%–61.4%)	59.9% (46.1%–71.2%)	25.4% (9.5%–45.1%)	37.0% (22.1%–52.0%)	56.3% (27.2%–77.6%)

Abbreviations: OS, overall survival; CBDCA, carboplatin; PAC, paclitaxel; CDDP, cisplatin; VNR, vinorelbine; DTX, docetaxel; S-1, tegafur/gimeracil/oteracil; CRT, chemoradiotherapy; CI, confidence interval; NR, not reached.

†Kaplan-Meier estimated values.

FIGURE S4

B

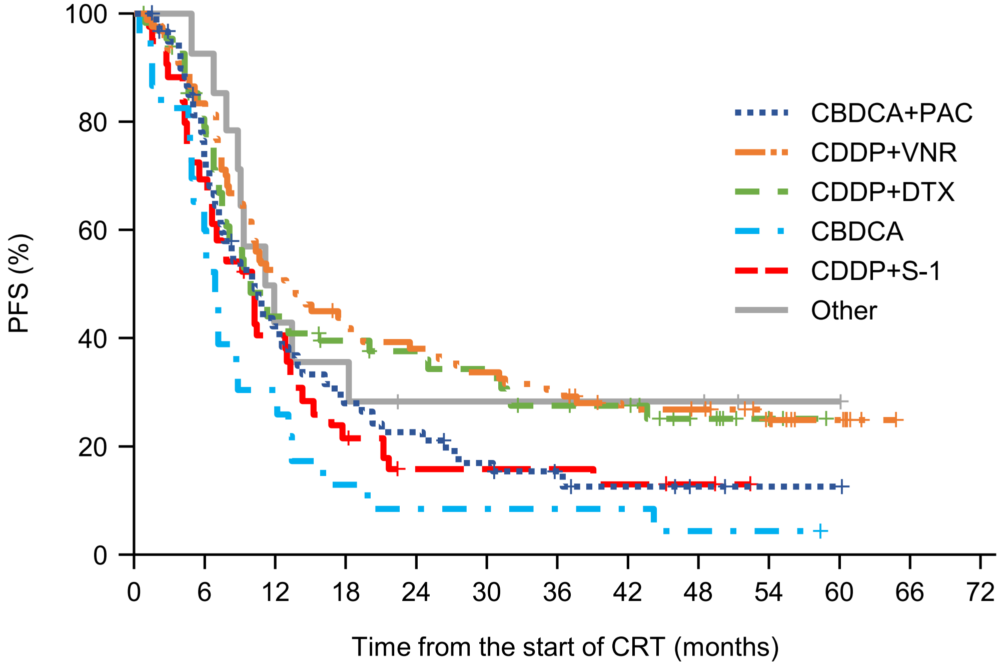

N at risk

CBDCA+PAC	62	43	24	16	13	9	6	4	2	1	1	0	0
CDDP+VNR	92	76	47	39	34	30	27	21	19	12	6	0	0
CDDP+DTX	70	52	29	24	22	20	15	14	7	3	0	0	0
CBDCA	23	14	7	3	2	2	2	2	1	1	0	0	0
CDDP+S-1	44	31	17	9	5	5	5	4	3	0	0	0	0
Other	14	13	6	5	3	3	3	3	3	1	1	0	0

	**CBDCA+PAC** n=62	**CDDP+VNR** n=92	**CDDP+DTX** n=71	**CBDCA** n=23	**CDDP+S-1** n=44	**Other** n=14
**Median PFS**[†] **(95% CI)**	10.2 months (7.2–13.1)	12.9 months (10.0–19.5)	9.9 months (7.6–19.9)	6.8 months (4.8–8.8)	10.0 months (6.2–12.9)	11.5 months (7.8–NR)
PFS at 12 months[†] (95% CI)	42.1% (29.3%–54.3%)	51.7% (41.0%–61.3%)	44.2% (32.1%–55.7%)	30.4% (13.5%–49.3%)	40.4% (25.9%–54.4%)	42.9% (17.7%–66.0%)
PFS at 24 months[†] (95% CI)	22.8% (13.0%–34.2%)	38.3% (28.4%–48.2%)	37.9% (26.3%–49.5%)	8.7% (1.5%–24.2%)	16.0% (6.8%–28.7%)	28.6% (8.8%–52.4%)
PFS at 36 months[†] (95% CI)	15.3% (7.4%–25.9%)	30.4% (21.3%–40.0%)	27.6% (17.2%–39.0%)	8.7% (1.5%–24.2%)	16.0% (6.8%–28.7%)	28.6% (8.8%–52.4%)

Abbreviations: PFS, progression-free survival; CBDCA, carboplatin; PAC, paclitaxel; CDDP, cisplatin; VNR, vinorelbine; DTX, docetaxel; S-1, tegafur/gimeracil/oteracil; CRT, chemoradiotherapy; CI, confidence interval; NR, not reached.

[†]Kaplan-Meier estimated values.

FIGURE S5 Kaplan–Meier plots of overall survival (A) and progression-free survival (B) according to consolidation therapy measured from the start of chemoradiotherapy in the full analysis set

A

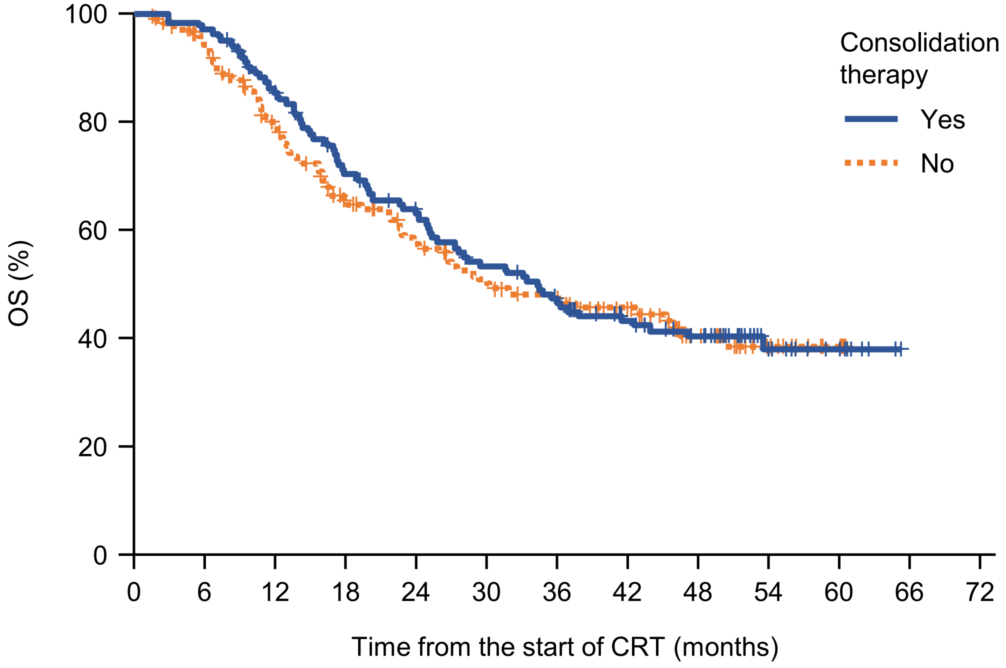

N at risk

Yes	148	144	123	98	85	71	61	46	36	15	8	0	0
No	158	141	113	86	71	59	52	43	24	13	5	0	0

Consolidation therapy	n	Median OS† (95% CI)	OS at 24 months† (95% CI)	OS at 36 months† (95% CI)	HR (95% CI)
Yes	148	34.2 months (25.7–44.0)	63.4% (54.8%–70.7%)	47.5% (39.0%–55.6%)	0.933 (0.684–1.273)
No	158	30.1 months (23.1–46.6)	57.8% (49.0%–65.6%)	47.6% (38.7%–55.9%)	

Abbreviations: OS, overall survival; CRT, chemoradiotherapy; CI, confidence interval; HR, hazard ratio.
†Kaplan-Meier estimated values.

FIGURE S5

B

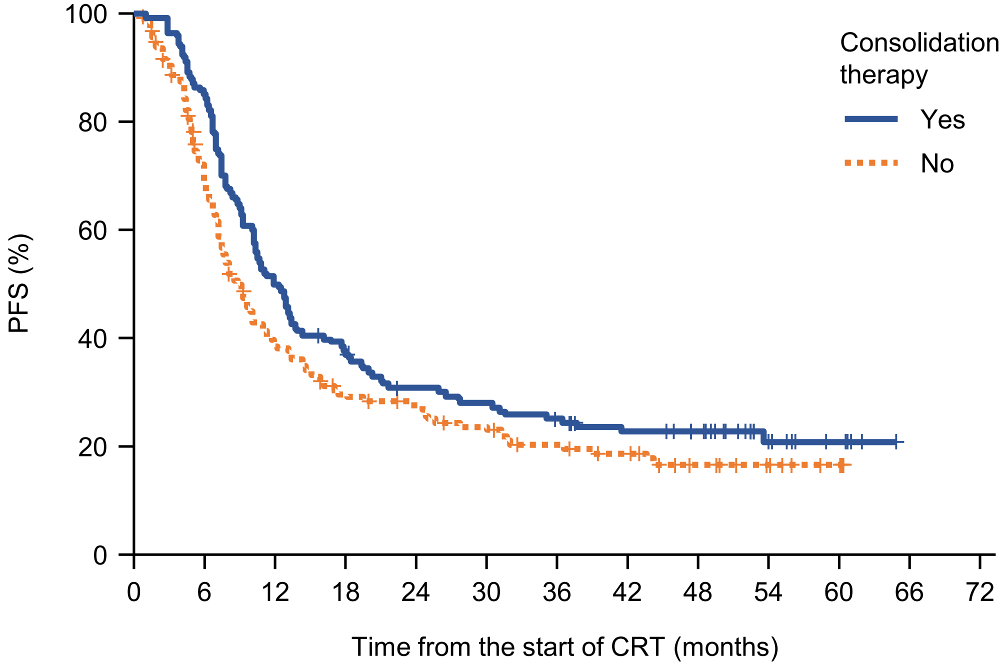

N at risk													
Yes	148	126	74	55	43	39	34	28	24	11	5	0	0
No	157	103	56	41	36	30	24	20	11	7	3	0	0

Consolidation therapy	n	Median PFS† (95% CI)	PFS at 24 months† (95% CI)	PFS at 36 months† (95% CI)	HR (95% CI)
Yes	148	12.1 months (10.2–13.7)	30.8% (23.5%–38.4%)	25.1% (18.3%–32.4%)	0.778 (0.602–1.006)
No	158	9.0 months (7.3–10.9)	27.6% (20.6%–35.0%)	20.5% (14.2%–27.6%)	

Abbreviations: PFS, progression-free survival; CRT, chemoradiotherapy; CI, confidence interval; HR, hazard ratio.
†Kaplan-Meier estimated values.

FIGURE S6 Timing of onset and cumulative incidence rate of pneumonitis, esophagitis, radiation dermatitis, and pericarditis in the full analysis set. The grade of pneumonitis is also indicated. Timing of onset was measured from the start of CRT

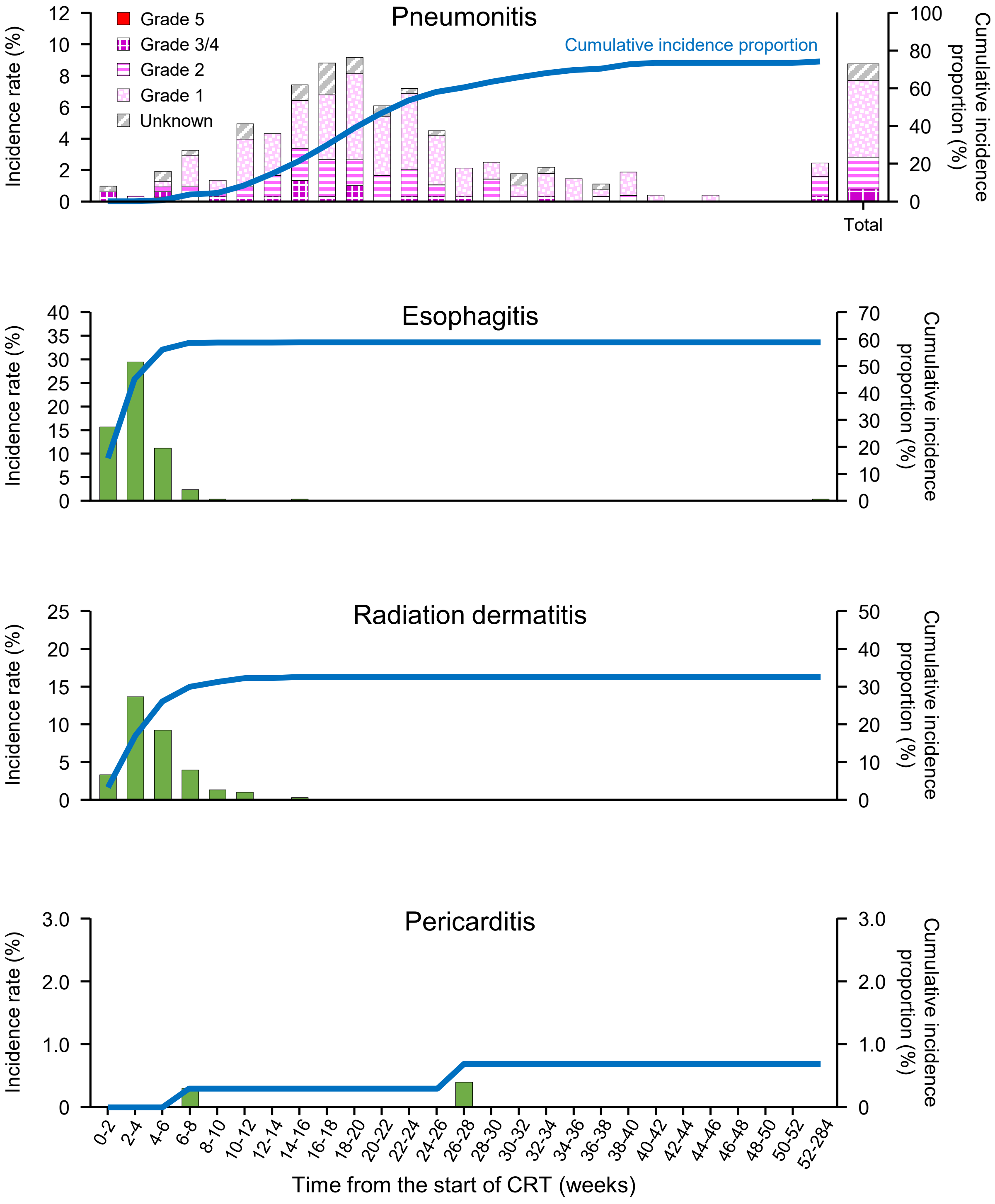

Abbreviations: CRT, chemoradiotherapy

FIGURE S7 Incidence rate of pneumonitis according to the type of chemotherapy received in the full analysis set (A) and m-sub (B)

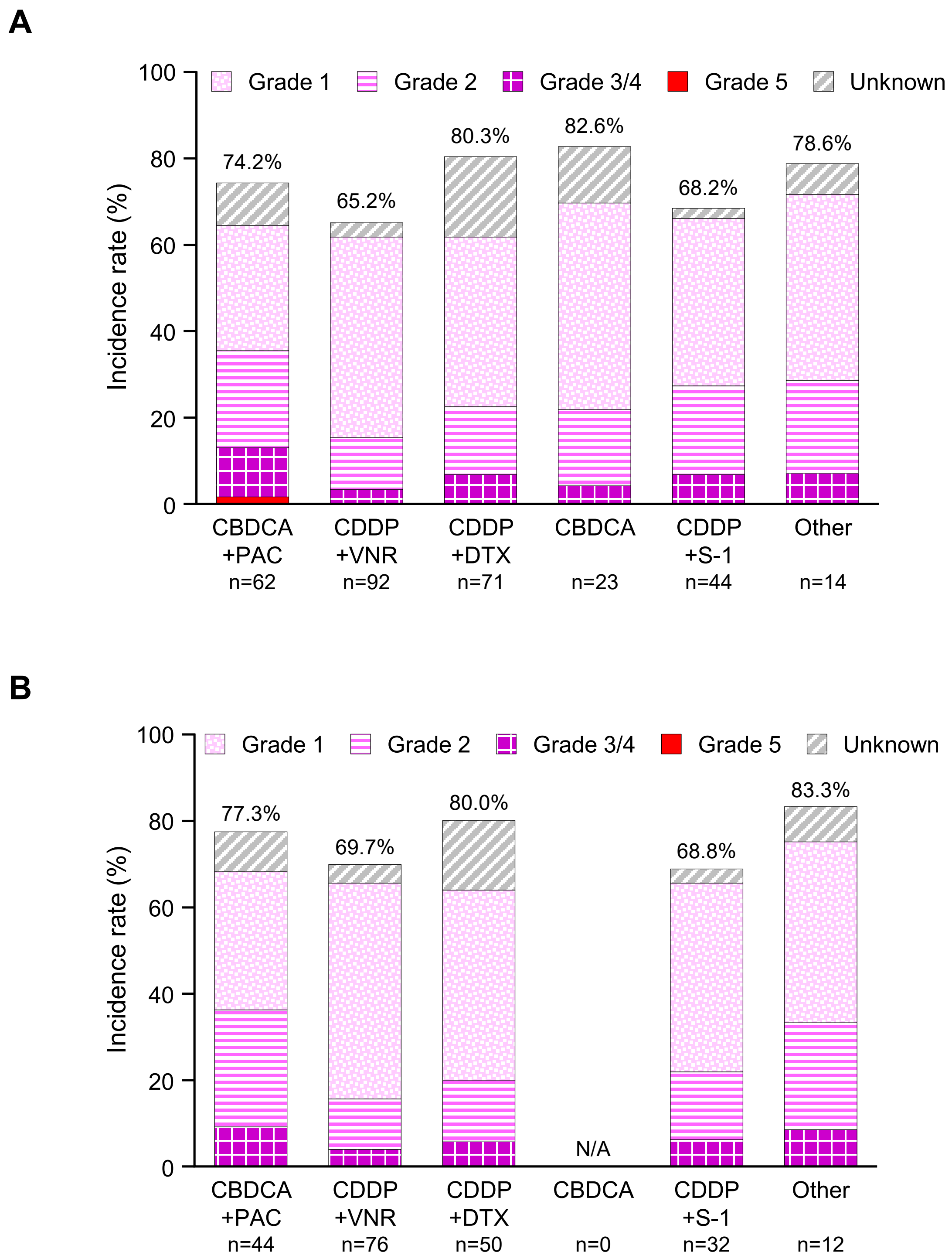

Abbreviations: m-sub, subgroup of patients deemed eligible for maintenance therapy after CRT; CBDCA, carboplatin; PAC, paclitaxel; CDDP, cisplatin; VNR, vinorelbine; DTX, docetaxel; S-1, tegafur/gimeracil/oteracil; CRT, chemoradiotherapy.
