# Supplementary material for: Real‐world outcomes of chemoradiotherapy for unresectable Stage III non‐small cell lung cancer: The SOLUTION study
Source: Cancer Med. 2020 Jul 30;9(18):6597–608. doi: 10.1002/cam4.3306 (PMC7520333; doi:10.1002/cam4.3306)
Supplement: Supplementary file 2 — Table S1‐S5 [file CAM4-9-6597-s002.docx]

**TABLE S1** Patient characteristics in the full analysis set (N = 306)

|  | **Value** |  | **Value** |
| --- | --- | --- | --- |
| Age, years, median (range) | 66.0 (37–84) | Primary lesion location |  |
| Sex |  | Right upper lobe | 137 (44.8%) |
| Male | 245 (80.1%) | Right middle lobe | 11 (3.6%) |
| Female | 61 (19.9%) | Right lower lobe | 46 (15.0%) |
| Smoking history |  | Left upper lobe | 88 (28.8%) |
| Current | 93 (30.4%) | Left lower lobe | 27 (8.8%) |
| Past | 180 (58.8%) | %VC | n = 271 |
| Never | 33 (10.8%) | Mean (SD) | 98.5 (18.3) |
| ECOG PS |  | <80 | 40 (13.1%) |
| 0 | 182 (59.5%) | ≥80 | 231 (75.5%) |
| 1 | 110 (35.9%) | FEV_1_/FVC, % | n = 271 |
| 2 | 10 (3.3%) | Mean (SD) | 70.4 (10.8) |
| 3/4 | 1 (0.3%) | <70 | 123 (40.2%) |
| Comorbidities |  | ≥70 | 148 (48.4%) |
| Yes | 225 (73.5%) | %DLco | n = 77 |
| Type of comorbidity |  | Mean (SD) | 87.9 (23.6) |
| COPD | 53 (17.3%) | <70 | 21 (6.9%) |
| Autoimmune disease | 4 (1.3%) | ≥70 | 56 (18.3%) |
| ILD | 2 (0.7%) | SpO_2_, % | n = 298 |
| IPF | 0 (0%) | Mean (SD) | 97.0 (1.6) |
| Non-IPF | 2 (0.7%) | <90 | 0 (0%) |
| Other | 205 (67.0%) | ≥90 | 298 (97.4%) |
| Stage |  | Reason for terminating first-line RT |  |
| IIIA | 159 (52.0%) | Completed as planned | 291 (95.1%) |
| IIIB | 147 (48.0%) | PD | 1 (0.3%) |
| Histological type |  | Toxicity | 2 (0.7%) |
| Adenocarcinoma | 144 (47.1%) | Other | 8 (2.6%) |
| Squamous cell carcinoma | 127 (41.5%) | Follow-up period, days |  |
| Neuroendocrine tumor | 9 (2.9%) | Mean (SD) | 932.2 (550.6) |
| Other | 25 (8.2%) | Median (range) | 853.0 (57–2014) |

Abbreviations: ECOG, Eastern Cooperative Oncology Group; PS, performance status; COPD, chronic obstructive pulmonary disease; ILD, interstitial lung disease; IPF, idiopathic pulmonary fibrosis; %VC, percent of vital capacity; SD, standard deviation; FEV_1_, forced expiratory volume in 1 second; FVC, forced vital capacity; %DLco, percent of diffusion capacity; SpO_2_, oxygen saturation; RT, radiotherapy; PD, progressive disease.

Values are number (%) of patients unless otherwise stated.

**TABLE S2** First-line treatment regimens in the full analysis set (N = 306)

| **Chemotherapy** | **Value**^†^ | **RT** | **Value** |
| --- | --- | --- | --- |
| Cisplatin + vinorelbine | 92 (30.1%) | Dose | n = 302 |
| Cisplatin + docetaxel | 71 (23.2%) | Median (range), Gy | 60.0 (10.0–72.0) |
| Carboplatin + paclitaxel | 62 (20.3%) | <54 Gy | 11 (3.6%) |
| Cisplatin + S-1 | 44 (14.4%) | ≥54 to ≤66 Gy | 289 (94.4%) |
| Carboplatin | 23 (7.5%) | >66 Gy | 2 (0.7%) |
| Other | 14 (4.6%) | V20 | n = 297 |
| Carboplatin + S-1 | 8 (2.6%) | Median (range) | 23.20% (1.6%–41.6%) |
| Carboplatin + pemetrexed | 2 (0.7%) | <25% | 171 (55.9%) |
| Cisplatin + pemetrexed | 2 (0.7%) | ≥25% | 126 (41.2%) |
| Cisplatin + etoposide | 2 (0.7%) | <35% | 283 (92.5%) |
|  |  | ≥35% | 14 (4.6%) |
|  |  | V5 | n = 297 |
|  |  | Median (range) | 36.60% (4.0%–70.0%) |
|  |  | <65% | 295 (96.4%) |
|  |  | ≥65% | 2 (0.7%) |

Abbreviations: S-1, tegafur/gimeracil/oteracil; RT, radiotherapy; V20, volume of lung that received a dose of ≥20 Gy; V5, volume of lung that received a dose of ≥5 Gy.

^†^Values are number (percent) of patients who received each chemotherapy regimen in the total population.

**TABLE S3** Risk factors for the onset of pneumonitis (full analysis set)

|  |  | **N** | **Pneumonitis, n (%)** | | **HR** | **95% CI** |
| --- | --- | --- | --- | --- | --- | --- |
| Age (years) | ≥20 to <65 | 134 | 93 | (69.4%) | - | - |
|  | ≥65 | 172 | 130 | (75.6%) | 1.175 | 0.900–1.534 |
|  | ≥20 to <75 | 275 | 198 | (72.0%) | - | - |
|  | ≥75 | 31 | 25 | (80.6%) | 1.447 | 0.954–2.196 |
| Sex | Male | 245 | 178 | (72.7%) | - | - |
|  | Female | 61 | 45 | (73.8%) | 0.924 | 0.666–1.282 |
| Smoking history | Current smoker | 93 | 61 | (65.6%) | 0.728 | 0.542–0.978 |
|  | Other than current smoker | 213 | 162 | (76.1%) | - | - |
| %VC | <80 | 40 | 27 | (67.5%) | 0.972 | 0.648–1.460 |
|  | ≥80 | 231 | 169 | (73.2%) | - | - |
| FEV1/FVC, % | <70 | 123 | 89 | (72.4%) | 0.918 | 0.693–1.217 |
|  | ≥70 | 148 | 107 | (72.3%) | - | - |
| %DLco | <70 | 21 | 14 | (66.7%) | 0.890 | 0.484–1.636 |
|  | ≥70 | 56 | 40 | (71.4%) | - | - |
| SpO_2_, % | <90 | 0 | 0 | - | - | - |
|  | ≥90 | 298 | 217 | (72.8%) | - | - |
| Stage | IIIA | 159 | 116 | (73.0%) | - | - |
|  | IIIB | 147 | 107 | (72.8%) | 0.988 | 0.760–1.286 |
| c-TNM, T-stage | T1-2 | 136 | 106 | (77.9%) | - | - |
|  | T3 | 60 | 43 | (71.7%) | 0.810 | 0.568–1.154 |
|  | T4 | 106 | 71 | (67.0%) | 0.812 | 0.601–1.096 |
| c-TNM, N-stage | N0-1 | 52 | 34 | (65.4%) | - | - |
|  | N2 | 147 | 113 | (76.9%) | 1.311 | 0.893–1.924 |
|  | N3 | 105 | 74 | (70.5%) | 1.134 | 0.755–1.702 |
| c-stage | Stage IA | 0 | 0 | - | - | - |
|  | Stage IB | 0 | 0 | - | - | - |
|  | Stage IIA | 0 | 0 | - | - | - |
|  | Stage IIB | 0 | 0 | - | - | - |
|  | Stage IIIA | 157 | 114 | (72.6%) | - | - |
|  | Stage IIIB | 145 | 106 | (73.1%) | 1.010 | 0.775–1.316 |
| p-TNM, T-stage | T1-2 | 2 | 2 | (100.0%) | - | - |
|  | T3 | 0 | 0 | - | - | - |
|  | T4 | 1 | 1 | (100.0%) | - | - |
| p-TNM, N-stage | N0-1 | 1 | 1 | (100.0%) | - | - |
|  | N2 | 1 | 1 | (100.0%) | - | - |
|  | N3 | 1 | 1 | (100.0%) | - | - |
| p-stage | Stage IA | 0 | 0 | - | - | - |
|  | Stage IB | 0 | 0 | - | - | - |
|  | Stage IIA | 0 | 0 | - | - | - |
|  | Stage IIB | 0 | 0 | - | - | - |
|  | Stage IIIA | 2 | 2 | (100.0%) | - | - |
|  | Stage IIIB | 1 | 1 | (100.0%) | - | - |
| Histologic type | Squamous cell carcinoma | 127 | 95 | (74.8%) | - | - |
|  | Other than squamous cell carcinoma | 178 | 127 | (71.3%) | 0.794 | 0.608–1.036 |
| Primary tumor site | Right upper lobe | 137 | 99 | (72.3%) | 1.014 | 0.779–1.321 |
|  | Right middle lobe | 11 | 9 | (81.8%) | 1.161 | 0.596–2.263 |
|  | Right lower lobe | 46 | 39 | (84.8%) | 1.443 | 1.021–2.040 |
|  | Left upper lobe | 88 | 59 | (67.0%) | 0.812 | 0.603–1.093 |
|  | Left lower lobe | 27 | 20 | (74.1%) | 0.932 | 0.589–1.475 |
| ECOG PS | 0 | 182 | 141 | (77.5%) | - | - |
|  | 1 | 110 | 74 | (67.3%) | 0.816 | 0.616–1.081 |
|  | ≥2 | 11 | 6 | (54.5%) | 0.535 | 0.236–1.211 |
| Comorbidities | Yes | 225 | 165 | (73.3%) | 1.045 | 0.775–1.410 |
|  | No | 81 | 58 | (71.6%) | - | - |
| Type of comorbidity | COPD | 53 | 41 | (77.4%) | 1.168 | 0.783–1.744 |
|  | Autoimmune disease | 4 | 2 | (50.0%) | 0.761 | 0.186–3.117 |
|  | ILD | 2 | 1 | (50.0%) | 0.562 | 0.077–4.082 |
|  | IPF | 0 | 0 | - | - | - |
|  | Non-IPF | 2 | 1 | (50.0%) | 0.562 | 0.077–4.082 |
|  | Unknown | 0 | 0 | - | - | - |
|  | Other | 205 | 147 | (71.7%) | 1.012 | 0.747–1.372 |
| First-line RT V20 | <25% | 171 | 115 | (67.3%) | - | - |
|  | ≥25%  <35%  ≥35% | 126  283  14 | 102  206  11 | (81.0%)  (72.8%)  (78.6%) | 1.406  -  1.806 | 1.076–1.836  -  0.984–3.315 |
| First-line RT V5 | <65% | 295 | 215 | (72.9%) | - | - |
|  | ≥65% | 2 | 2 | (100.0%) | 4.953 | 1.212–20.236 |
| Reason for ending first-line RT | As planned | 291 | 215 | (73.9%) | - | - |
|  | Other than planned | 11 | 6 | (54.5%) | 0.659 | 0.293–1.485 |
| First-line RT dose | <54 Gy | 11 | 4 | (36.4%) | 0.355 | 0.132–0.955 |
|  | ≥54 to ≤66 Gy | 289 | 215 | (74.4%) | - | - |
|  | >66 Gy | 2 | 2 | (100.0%) | 1.957 | 0.486–7.890 |

Abbreviations: HR, hazard ratio; CI, confidence interval; %VC, percent of vital capacity; FEV1, forced expiratory volume in 1 second; FVC, forced vital capacity; %DLco, percent of diffusion capacity; SpO_2_, oxygen saturation; ECOG, Eastern Cooperative Oncology Group; PS, performance status; COPD, chronic obstructive pulmonary disease; ILD, interstitial lung disease; IPF, idiopathic pulmonary fibrosis; RT, radiotherapy; V20, volume of lung that received a dose of ≥20 Gy; V5, volume of lung that received a dose of ≥5 Gy.

**TABLE S4** Adverse events in ≥5% of patients in the full analysis set (N = 306) according to chemotherapy regimen

|  | CBDCA+PAC (n = 62) | CDDP+VNR (n = 92) | CDDP+DTX (n = 71) | CBDCA (n = 23) | CDDP+S-1 (n = 44) | Other  (n = 14) |
| --- | --- | --- | --- | --- | --- | --- |
| Any AE | 62 (100.0%) | 90 (97.8%) | 70 (98.6%) | 23 (100.0%) | 44 (100.0%) | 14 (100.0%) |
| Pneumonitis | 46 (74.2%) | 60 (65.2%) | 57 (80.3%) | 19 (82.6%) | 30 (68.2%) | 11 (78.6%) |
| Esophagitis | 48 (77.4%) | 54 (58.7%) | 39 (54.9%) | 14 (60.9%) | 24 (54.5%) | 3 (21.4%) |
| White blood cell count decreased | 32 (51.6%) | 51 (55.4%) | 32 (45.1%) | 6 (26.1%) | 23 (52.3%) | 3 (21.4%) |
| Decreased appetite | 11 (17.7%) | 40 (43.5%) | 25 (35.2%) | 7 (30.4%) | 21 (47.7%) | 4 (28.6%) |
| Radiation dermatitis | 20 (32.3%) | 25 (27.2%) | 19 (26.8%) | 11 (47.8%) | 21 (47.7%) | 4 (28.6%) |
| Nausea | 6 (9.7%) | 39 (42.4%) | 18 (25.4%) | 3 (13.0%) | 18 (40.9%) | 3 (21.4%) |
| Constipation | 15 (24.2%) | 21 (22.8%) | 9 (12.7%) | 4 (17.4%) | 21 (47.7%) | 2 (14.3%) |
| Malaise | 5 (8.1%) | 32 (34.8%) | 18 (25.4%) | 4 (17.4%) | 12 (27.3%) | 0 (0%) |
| Neutrophil count decreased | 9 (14.5%) | 25 (27.2%) | 17 (23.9%) | 3 (13.0%) | 12 (27.3%) | 2 (14.3%) |
| Platelet count decreased | 12 (19.4%) | 3 (3.3%) | 5 (7.0%) | 8 (34.8%) | 19 (43.2%) | 5 (35.7%) |
| Anemia | 9 (14.5%) | 13 (14.1%) | 9 (12.7%) | 1 (4.3%) | 16 (36.4%) | 3 (21.4%) |
| Pyrexia | 7 (11.3%) | 11 (12.0%) | 14 (19.7%) | 2 (8.7%) | 8 (18.2%) | 3 (21.4%) |
| Diarrhea | 2 (3.2%) | 7 (7.6%) | 21 (29.6%) | 0 (0%) | 9 (20.5%) | 1 (7.1%) |
| Hiccups | 2 (3.2%) | 11 (12.0%) | 12 (16.9%) | 0 (0%) | 13 (29.5%) | 1 (7.1%) |
| Vomiting | 6 (9.7%) | 7 (7.6%) | 3 (4.2%) | 0 (0%) | 7 (15.9%) | 0 (0%) |
| Stomatitis | 0 (0%) | 9 (9.8%) | 3 (4.2%) | 0 (0%) | 4 (9.1%) | 0 (0%) |
| Alopecia | 3 (4.8%) | 9 (9.8%) | 4 (5.6%) | 0 (0%) | 0 (0%) | 0 (0%) |

Abbreviations: CBDCA, carboplatin; PAC, paclitaxel; CDDP, cisplatin; VNR, vinorelbine; DTX, docetaxel; S-1, tegafur/gimeracil/oteracil; AE, adverse event.

Values are number (percent) of patients.

## **TABLE S5** Adverse events in ≥5% of patients in the full analysis set (N = 306) according to use of consolidation chemotherapy

|  | With consolidation  therapy  (n = 148) | Without consolidation therapy  (n = 158) |
| --- | --- | --- |
| Total | 146 (98.6%) | 157 (99.4%) |
| Pneumonitis | 108 (73.0%) | 115 (72.8%) |
| Esophagitis | 84 (56.8%) | 98 (62.0%) |
| White blood cell count decreased | 80 (54.1%) | 67 (42.4%) |
| Decreased appetite | 61 (41.2%) | 47 (29.7%) |
| Radiation dermatitis | 47 (31.8%) | 53 (33.5%) |
| Nausea | 53 (35.8%) | 34 (21.5%) |
| Constipation | 48 (32.4%) | 24 (15.2%) |
| Malaise | 39 (26.4%) | 32 (20.3%) |
| Neutrophil count decreased | 34 (23.0%) | 34 (21.5%) |
| Platelet count decreased | 26 (17.6%) | 26 (16.5%) |
| Anemia | 27 (18.2%) | 24 (15.2%) |
| Pyrexia | 17 (11.5%) | 28 (17.7%) |
| Diarrhea | 14 (9.5%) | 26 (16.5%) |
| Hiccups | 24 (16.2%) | 15 (9.5%) |
| Vomiting | 17 (11.5%) | 6 (3.8%) |
| Stomatitis | 6 (4.1%) | 10 (6.3%) |
| Alopecia | 13 (8.8%) | 3 (1.9%) |

Values are number (percent) of patients.
